# Development of Antipsychotic Medications with Novel Mechanisms of Action Based on Computational Modeling of Hippocampal Neuropathology

**DOI:** 10.1371/journal.pone.0058607

**Published:** 2013-03-19

**Authors:** Peter J. Siekmeier, David P. vanMaanen

**Affiliations:** 1 Laboratory for Computational Neuroscience, McLean Hospital, Belmont, Massachusetts, United States of America; 2 Harvard Medical School, Boston, Massachusetts, United States of America; Tulane University Medical School, United States of America

## Abstract

A large number of cellular level abnormalities have been identified in the hippocampus of schizophrenic subjects. Nonetheless, it remains uncertain how these pathologies interact at a system level to create clinical symptoms, and this has hindered the development of more effective antipsychotic medications. Using a 72-processor supercomputer, we created a tissue level hippocampal simulation, featuring multicompartmental neuron models with multiple ion channel subtypes and synaptic channels with realistic temporal dynamics. As an index of the schizophrenic phenotype, we used the specific inability of the model to attune to 40 Hz (gamma band) stimulation, a well-characterized abnormality in schizophrenia. We examined several possible combinations of putatively schizophrenogenic cellular lesions by systematically varying model parameters representing NMDA channel function, dendritic spine density, and GABA system integrity, conducting 910 trials in total. Two discrete “clusters” of neuropathological changes were identified. The most robust was characterized by co-occurring modest reductions in NMDA system function (-30%) and dendritic spine density (-30%). Another set of lesions had greater NMDA hypofunction along with low level GABA system dysregulation. To the schizophrenic model, we applied the effects of 1,500 virtual medications, which were implemented by varying five model parameters, independently, in a graded manner; the effects of known drugs were also applied. The simulation accurately distinguished agents that are known to lack clinical efficacy, and identified novel mechanisms (e.g., decrease in AMPA conductance decay time constant, increase in projection strength of calretinin-positive interneurons) and combinations of mechanisms that could re-equilibrate model behavior. These findings shed light on the mechanistic links between schizophrenic neuropathology and the gamma band oscillatory abnormalities observed in the illness. As such, they generate specific falsifiable hypotheses, which can guide postmortem and other laboratory research. Significantly, this work also suggests specific non-obvious targets for potential pharmacologic agents.

## Introduction

Schizophrenia is a debilitating, lifelong illness affecting approximately 1% of the population worldwide [1]. Beginning with Thorazine (chlorpromazine) in the 1950s, antipsychotic medications have been used to treat the condition. However, despite several years of research, and the introduction of a number of new agents, all currently used antipsychotics are far from ideal. They are capable of ameliorating some symptoms in many, though not all, schizophrenic patients, and none represents a cure to the disease. Moreover, these medications carry significant side effect burdens [2].

The absence of development of antipsychotic medications with fundamentally new mechanisms of action [3] stands in stark contrast to the vast amounts that have been learned over the past 20 years on the cellular level abnormalities associated with the disease. The hippocampal neurobiology of the illness has been the subject of a number of recent comprehensive and detailed reviews [4]. Broadly, studies on this and other brain areas have revealed: (1) Dysfunction in the gamma-aminobutyric acid (GABA) system. Deficiencies in GABAergic innervation have been seen, as a result of decreased number of particular subtypes of GABA neurons or GABAergic tone, and a (presumably compensatory) increase in postsynaptic GABA receptor expression [5]; (2) Glutamatergic system deficiency. This is manifested, for example, as decreased expression of N-methyl-D-aspartic acid (NMDA) receptors, and/or hypofunction of NMDA synaptic activity [6], [7]; and (3) Decreases in brain connectivity. Diminished dendritic spine density has been seen, for example, in postmortem and animal models of the illness [8], [9].

One reason this large and growing body of neurobiological knowledge has not translated into more effective treatments is that we do not have convincing causative links between cellular level abnormalities and particular symptoms or sets of symptoms. This is a problem that is characteristic of psychiatric illnesses in general, and stands in contrast to many other medical illnesses, in which, for example, the underlying genetic abnormality, the dysfunctional protein expressed, the function of this protein, and the manner in which this causes illness pathology are well understood. This is made particularly difficult because function in a given region, such as hippocampus, is likely an *emergent* phenomenon; it is extremely difficult to intuit the behavior of the overall system by looking at one, or even a few of its constituent cellular level behaviors or interactions in isolation [10]. It is difficult to imagine designing an effective intervention without taking this into account.

Oscillatory brain activity is an emergent, system level behavior that stands at an intermediate level of complexity between the cellular and the clinical. A large amount of recent research has indicated that schizophrenic patients show synchronization deficiencies in neural processing [11], particularly in the gamma frequency band [12]–[17]. Importantly, there is also evidence that gamma activity subserves particular cognitive functions, such as perceptual binding within a particular sensory modality, or integration of information from different sensory modalities [18], to form a coherent percept. Thus, disturbed function may be etiologically related to some of the positive symptoms of schizophrenia, such as hallucinations or compromised reality testing.

Given the complexity of schizophrenia, it is unsurprising that computational modeling has been applied in an attempt to better understand this illness and its possible etiology. While there are exceptions [19], [20], many have been abstract ANN (artificial neural network) style models, and they have tended to examine a single hypothetical pathology, such as connectivity disturbance [21], hyperdopaminergia [22], or deficient perforant path input into hippocampal formation [23]. One reason for this is that creating networks of biophysically detailed cellular models, and running large numbers of parameter assumptions (corresponding to different combinations of neural lesions or medication effects) are very computationally demanding undertakings. However, the development of computers with processing capacity several orders of magnitude greater than those of a generation ago now place us in a position such that we can begin to address these questions via “tissue level” computational work, and this is the approach we have taken here. Using a 72-processor supercomputer, we have created a biophysically detailed computational simulation of hippocampus, and use specific quantitative inability of the model to attune to 40 Hz stimulatory drive as a marker of the schizophrenic phenotype. We then introduce multiple putative schizophrenogenic cellular level abnormalities, as outlined above, into the model in a graded and combinatorial way, and find that two distinct “clusters” of pathologies can create the schizophrenic phenotype. Then, a large number of virtual medication effects are applied to the schizophrenic model, and combinations that return the model to its baseline (non-diseased) state are identified. The potentially ameliorative mechanisms identified are non-obvious, and do not represent simple reversals of the causative lesions.

## Methods

### Computational Model

The hippocampal model consists of 160 pyramidal cells and interneurons of three subtypes—30 basket cells, 30 chandelier cells, and 20 calretinin-positive (CR+), or interneuron projecting, cells. For pyramidal cells, we used the 64 compartment model described by Traub et al [24]. Interneuron models were based on the 46 compartment model of Traub and Miles [25]. Both include realistic dendritic arbors and incorporate 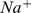, 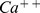, 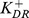
_,_ 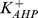,
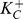
and
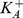
channels with Hodgkin-Huxley dynamics distributed along the somato-dendritic axis. Interneurons of different subclasses were defined by their axonal projections patterns, based the hippocampal model described by the author [26]. Full details of individual neuron models and their connectivity, a description of the manner in which a simulated EEG was calculated, and details of the model’s hardware implementation can be found in Text S1. Model parameters are given in online Tables S1, S2, S3, and S4.

### Implementation of Putative Schizophrenogenic Cellular Level Abnormalities

#### Glutamatergic system dysfunction:

A number of recent research studies have found decreased density of NMDA synapses in schizophrenic hippocampus and/or hypofunction of these synapses, and have quantified this effect. Tsai et al [27], in postmortem work, found a decrease of 37% in glutamate levels in hippocampus of schizophrenics. This group also found a 55% increase in N-acetylaspartylglutamic acid (NAAG) in this area; together with work by Bergeron et al [28] that demonstrated an inverse relationship between NAAG level and NMDA current, this suggests that schizophrenic patients may experience NMDA hypofunction via decreases in the conductance of the NMDA channel. Law and Deakin [29] found a decrease in the obligatory NMDAR1 subunit of the NMDA receptor of 40%. Similarly, Harrison et al [30], in postmortem work looking at markers of glutamate receptors in schizophrenic hippocampus, found a decrease of 26% in mRNA coding for NR1, a subunit of the NMDA receptor.

Thus, the research suggests a spectrum of possible NMDA deficits. To capture the full range of possible values, we implemented NMDA effects by decreasing maximum conductance (g_max_) of the model NMDA receptors by 0 to 45%, in increments of 5%. We also performed trials in which the number of NMDA receptors was decreased through this range, and found quantitatively similar effects (data not shown).

#### Connectivity disturbances:

A “pruning hypothesis” of schizophrenia has long been suggested [9], [31]. Much of the substantiation for this, however, came from indirect measurements (e.g., decreased neuropil volume). Studies have examined this quantitatively by looking at density of spines on neuronal dendrites. Law et al [32] found a decrease in levels of mRNA for spinophilin (a marker for dendritic spines) of 44.5%, on average. Garey et al [33], in a postmortem study Golgi staining, saw a decreased spine density in temporal lobe of patients of 59.4%. DeVito et al [34], using a genetic knockout model of NMDA receptor hypofunction (a serine racemase knockout mouse), found a decreased dendritic spine density of 40.5%. To capture the full range of possible values, we decreased pyramidal cell spine density from 0 to 60%, in increments of 5%.

#### GABA system dysregulation:

Heckers et al [35] found decreased expression of mRNA for two isoforms of the GABA-synthesizing enzyme glutamic acid decarboxylase GAD65 and GAD67 decreased by 14% and 4%, respectively, in schizophrenic hippocampi. Bird et al [36], in a postmortem study of brains of psychotic patients found GAD to be decreased by 48.2%. Fatemi et al [37] and Torrey et al [38] found decreases in reelin in schizophrenic hippocampus of 29% and 46%, respectively. However, other studies have found *increases* in GABA receptor binding. For example, Benes et al [39] showed increases of 45% to 82% depending on subfield of hippocampus. It is felt that this may represent a compensatory upregulation of postsynaptic GABA receptors, in response to decreased GABAergic activity [40].

To apply these changes, we simulated the decrease in GABAergic tone by decreasing the number of GABAergic projections (that is, projections from model interneurons) from 0 to 45% in increments of 7.5%; we simulated increased weight of postsynaptic GABA receptors by increasing the weight factor at these synapses from 0 to 60%. We made these changes in tandem, testing 7 “ordered pairs” of parameter values ([0, 0] to [-45%, +60%,]), where each element is [change in GABAergic tone, GABA postsynaptic weight change]. This was done because when searching large parameter spaces, adding an additional dimension increases the number of trials multiplicatively, and searching four dimensions for the current problem would have been prohibitively time consuming.

### Calculation of Illness Metric

We created a metric to quantify the “schizophrenic-ness” of a given model run, based on its quantitative similarity to the experimental findings of Teale et al [16]. This study was used because it employs a steady state evoked potential (SSEP) task, and detailed source localization carried out in the study revealed that the source of the oscillatory activity recorded was temporal lobes, and thus may be hippocampal in origin. Based on their data (their Figure 6, p. 1486), which shows 40 Hz oscillatory activity as a function of time when patients are receiving the stimulus, at maximum patients showed a decrease of approximately 26% at this frequency, compared with controls (this represents an average over left and right hemispheres). The many SSEP experiments that have been carried out on schizophrenic patients indicate that when exposed to 20 or 30 Hz stimulation, patients did not show a response significantly different from controls[41].

**Figure 1:**
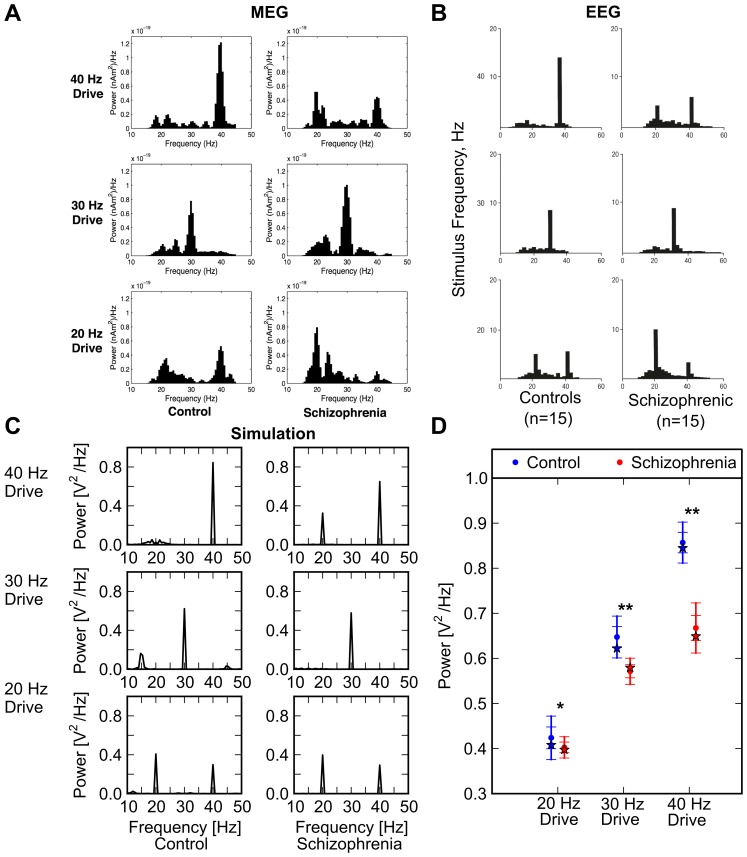
Brain oscillatory activity from clinical magnetoencephalographic (MEG) and EEG studies, and output of computational model. (A) Control subjects (left three histograms) and schizophrenic patients (right three histograms) were exposed to auditory click trains at 20, 30, and 40 Hz. Resultant MEG power spectra are shown (from Vierling-Claassen et al [54]). (B) The same experimental conditions as (A) above were used, but EEG activity was recorded (from Kwon et al [41]). (C) Simulated EEG power spectra from model when driven at 20, 30, and 40 Hz. Note correspondence with clinical data of panels (A) and (B). (This is the “primary point model”, as defined in Fig 2.) (D) Graph of power spectrum peaks from index schizophrenic patient of panel (C) plus 20 simulated patients (in red), and index control patient of panel (C) plus 20 simulated control subjects (blue). In all cases, index patient is indicated by a star; simulated patient averages are indicated by dot, and one and two standard deviations are shown by tick marks on error bar. Although computational model outcomes are not strictly analogous to data from clinical studies [59]-[61], we have calculated p-values, by convention (* p < 0.01, ** p < 0.001). Note that *Group* x *Frequency* interaction was highly significant, due to the fact that group differences were largest at 40 Hz; please see text for additional details of statistical analysis.

Therefore, for a model to said to be schizophrenic: (1) 20 and 30 Hz activity were required to be within a given tolerance of the baseline case. We used +/- 7.5% for this value, based on the standard deviation of our 20 simulated control patients (Figure 1); models that failed for either frequency were given a score of 0. (2) 40 Hz activity was required to be significantly decreased from the baseline condition. To quantify this, percentage decrease of schizophrenic condition vs. control condition was calculated (that is, [power of 40 Hz response, baseline condition] – [power of 40 Hz response, schizophrenic condition] / [power of 40 Hz response, baseline condition]). If this equaled 26%, the model received a score of 1; to the extent that this differed from 26%, in absolute value, the score was decreased. Calculation of the illness metric is presented formally in Text S1 and accompanying Table S5.

To ensure that the most highly schizophrenic case identified represented a robust model behavior, we created 20 simulated schizophrenic subjects and 20 control subjects. In order to create a simulated subject, we re-seeded the random number generator to generate a new model, and had it perform the experimental task. Thus, each simulated subject had different specific cell-to-cell pattern of connectivity, but the projection probabilities between cell types (as defined in Table S4) were identical. Using these data, we ran a mixed model ANOVA, entering *Group* (control, schizophrenic) as a between subjects factor and *Frequency* (20 Hz, 30 Hz, 40 Hz) as a repeated measures factor. To test the specificity of putative ANOVA findings, hierarchical regressions were run.

## Results

The first section below illustrates the network model’s ability to attune to 20, 30, and 40 Hz stimulation in the baseline condition. Subsequent sections show the results of implementation of schizophrenogenic cellular lesions, and the results of trials that incorporate the effect of both known medications and virtual antipsychotic drugs.

### Reproduction of Baseline Oscillatory Activity

After tuning, we drove the hippocampal model at 20, 30, and 40 Hz; a simulated EEG was generated for each and was analyzed via fast Fourier transform (FFT) to determine which frequencies were present. The model reproduced, in a quantitatively similar way, frequency behaviors shown in control subjects (Fig 1A [left panels] and 1B [left panels] experimental; Fig 1C [left panels] model output).

To confirm these model results, we created 20 simulated control subjects, as described in the Methods section. The results of these runs are shown in Figure 1D (blue points). It is clear that the behavior of our simulated index control subject is representative of the group of simulated controls, and that this group is similar to that of the control subjects of experimental studies.

### Effects of Putative Schizophrenogenic Cellular Level Abnormalities on Model Behavior

**Figure 2:**
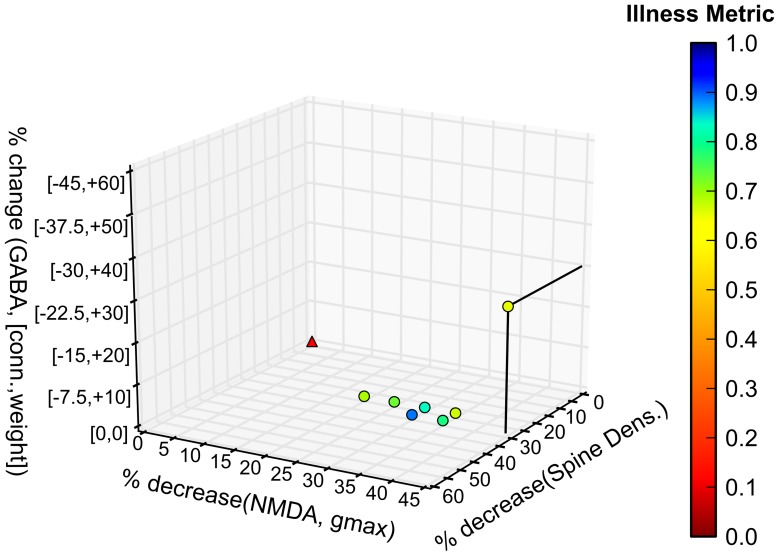
Model output showing unique combinations of abnormalities that may give rise to the schizophrenic phenotype. Degree of GABA system dysregulation, extent of NMDA hypofunction, and spine density decrease are shown on the axes, respectively. Origin (0, 0, 0) represents the control (unaffected) condition. The degree to which model outputs match experimental findings (illness metric) is indicated via color scale. All model outcomes with illness metric > 0.65 are shown.

The manner in which the cellular level pathology that has been observed in schizophrenic hippocampus was instantiated as parameter changes in the model is detailed in Methods. Briefly, decreased NMDA activity was operationalized by decreasing maximum conductance (g_max_) of the model NMDA receptors (in 10 increments); connectivity deficits were operationalized by decreasing pyramidal cell dendritic spine density (13 increments); and GABA system dysregulation was implemented by a joint decrease in GABA tone and increase in postsynaptic weight (7 increments). Iterations representing all possible levels of the aforementioned cellular level lesions were run: that is, we exhaustively searched the parameter space, running 10×13×7  =  910 iterations in total. Each iteration consisted of three trials; in each, the network was driven at a given frequency (20, 30, or 40 Hz), and a simulated EEG was written to file and was analyzed via fast Fourier transform (FFT) to determine which frequencies were present, and their relative power. The degree to which this matched the pattern seen in the clinical studies (i.e., the degree to which there was a specific deficit in 40 Hz response) was quantified using the illness metric, which ranged from 1 (most schizophrenic) to 0, as described in Methods. Figure 2 graphically depicts the results of these trials.

Clearly, a number of points produce schizophrenia-like results. There is a prominent cluster centered at a point characterized by an NMDA decrease of 30%, a spine density decrease of 30%, and a GABA deficit of 0 (which we will call the “primary point”). There is another point characterized by an NMDA decrease of 45%, a spine density decrease of 30%, and a GABA system defect of (–37.5, +30%), as defined in Methods (which we will call the “secondary point”). For the primary point, power spectra of oscillatory activity in response to 20, 30, and 40 Hz drive is shown in Fig 1C, in comparison with control behavior (Figs 1A, B). 40 Hz response is decreased to about 24% below the control case, calculated as an average of 20 simulated control patients; 20 and 30 Hz responses are roughly the same as those of controls. This again was confirmed by re-running the model with 20 simulated schizophrenic patients.

To more formally test these effects, we ran a *Group* (control, schizophrenic) x *Frequency* (20 Hz, 30 Hz, 40 Hz) ANOVA. Both the main effects of *Frequency* (F [2, 80]  =  4812.6, p < 0.001, Greenhouse-Geisser correction: ε  =  0.87) and *Group* (F [2, 80]  =  289.05, p < 0.001, ε  =  0.87) were significant. Critically, these effects were qualified by a significant *Group* by *Frequency* interaction, driven by greatest group differences at 40 Hz (see Fig 1D). Because groups differed in all three frequencies, a set of hierarchical regression analyses was run to test the specificity of the findings. Specifically, in the first regression, we entered power at 20 and 30 Hz in the first step, and *Group* (dummy-coded) in the second step, in order to predict power at 40 Hz. The model was significant, indicating that *Group* predicted 40 Hz activity when controlling for power at 20 and 30 Hz (ΔR^2^  =  0.101, ΔF [1], [38]  =  77.64, p < 0.001). Critically, when entering 40 in the first step, *Group* predicted neither 30 Hz power (ΔR^2^  =  0.003, ΔF [1], [38]  =  0.81, p  =  0.375) nor 20 Hz power (ΔR^2^  =  0.025, ΔF [1], [38]  =  0.81, p  =  0.193). Thus, group differences were specific to 40 Hz.

**Figure 3:**
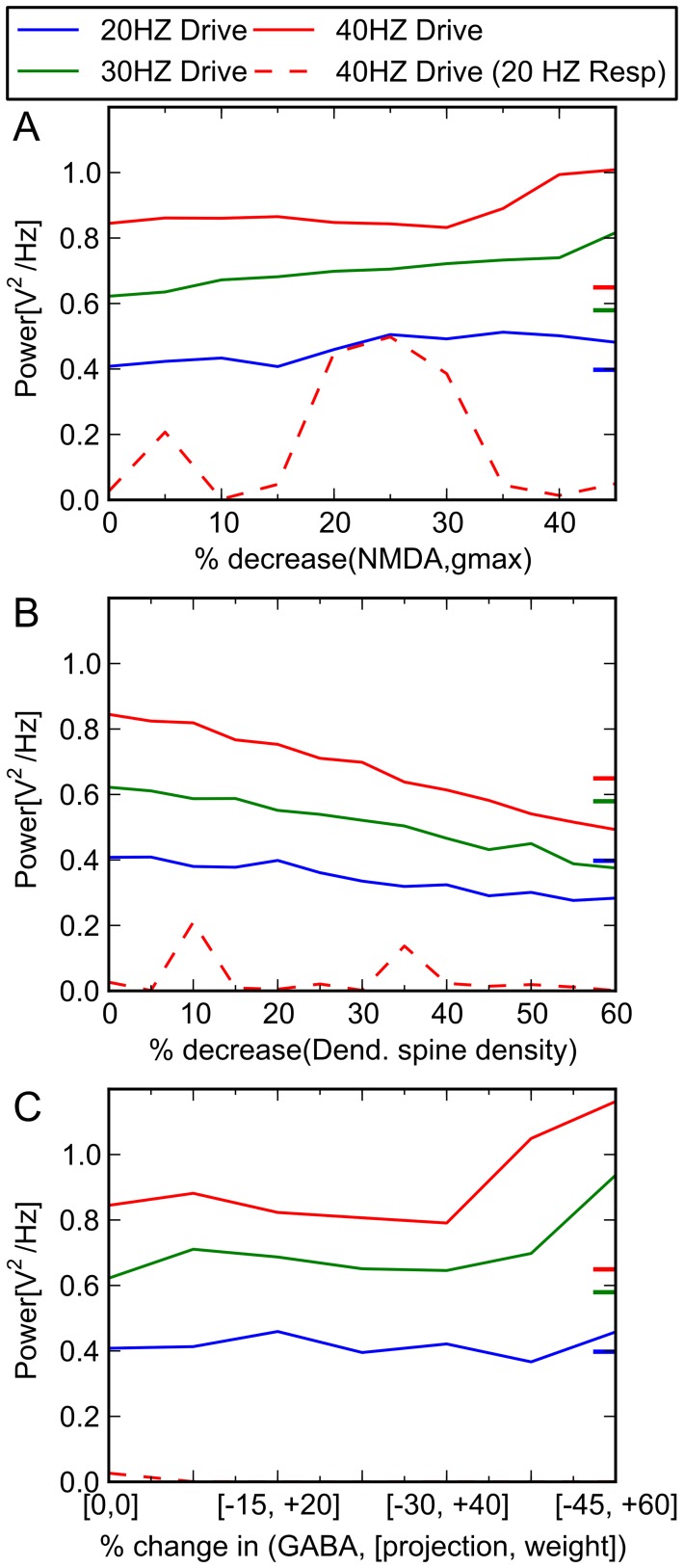
Response of system with respect to change in single parameters. Oscillatory activity (power) at 20, 30, and 40 Hz with respect to (A) decreased NMDA activity, (B) decreased pyramidal cell spine density, and (C) increasing GABA defect is shown. Colored tick marks on right border of graphs indicate oscillatory behavior characteristic of schizophrenic patients. Solid lines represent model response at that frequency to drive at the given frequency (e.g., solid blue line represents power of 20 Hz activity when model is driven at 20 Hz). Dashed red line represents 20 Hz response to 40 Hz drive. Resp  =  response.

**Figure 4:**
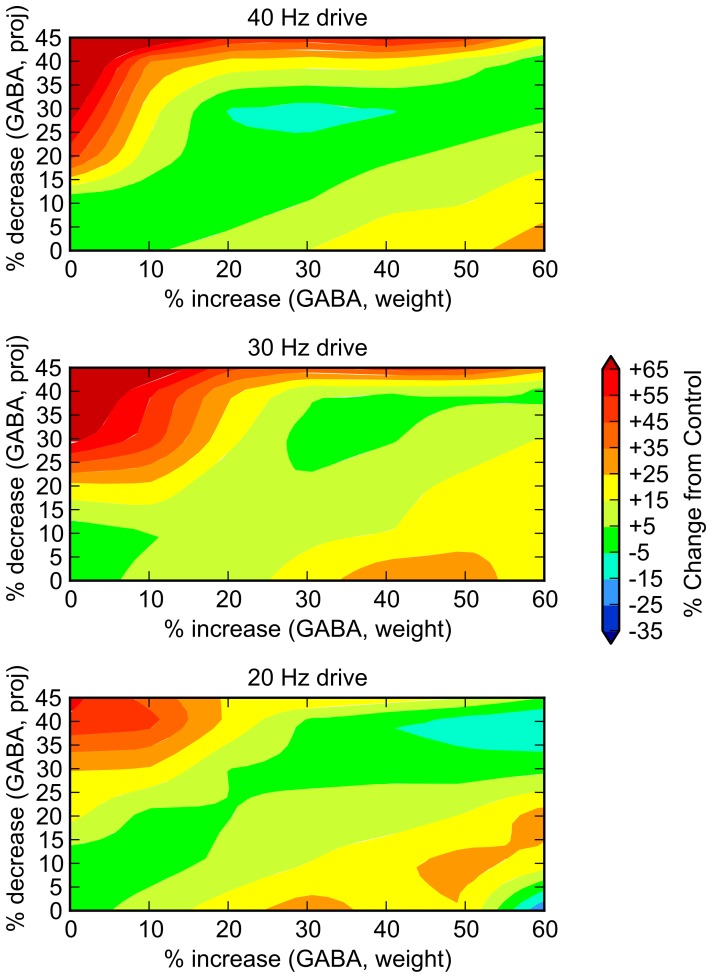
Relative contribution of each component of GABA system deficit. To understand the relative contribution of each component of the GABA deficit, we performed 7×7  =  49 trials, varying the GABA projection parameter (y axis) and the postsynaptic weight parameter (x axis) independently through ranges of 0 to –45% and 0 to +60%, respectively. System response at 20, 30, and 40 Hz drive are shown. While it appears that joint increases in these parameters (i.e., a diagonal extending from the origin) show some preferential decrease in 40 Hz behavior, it is clear that no path through the 2D space is significantly schizophrenia-like. Dark green indicates areas in which there is minimal change (+/- 5%) from control.

In an attempt to understand the relative contributions of each of these neural level abnormalities individually to the functioning of the system, we performed a “partial derivative” analysis for each. That is, we examined the overall behavior of the system in response to one lesion at a time, holding the others constant. The results are shown in Figures 3 and 4. Significantly, no single abnormality alone accounts for the findings.

### Analysis of Oscillatory Dynamics

**Figure 5:**
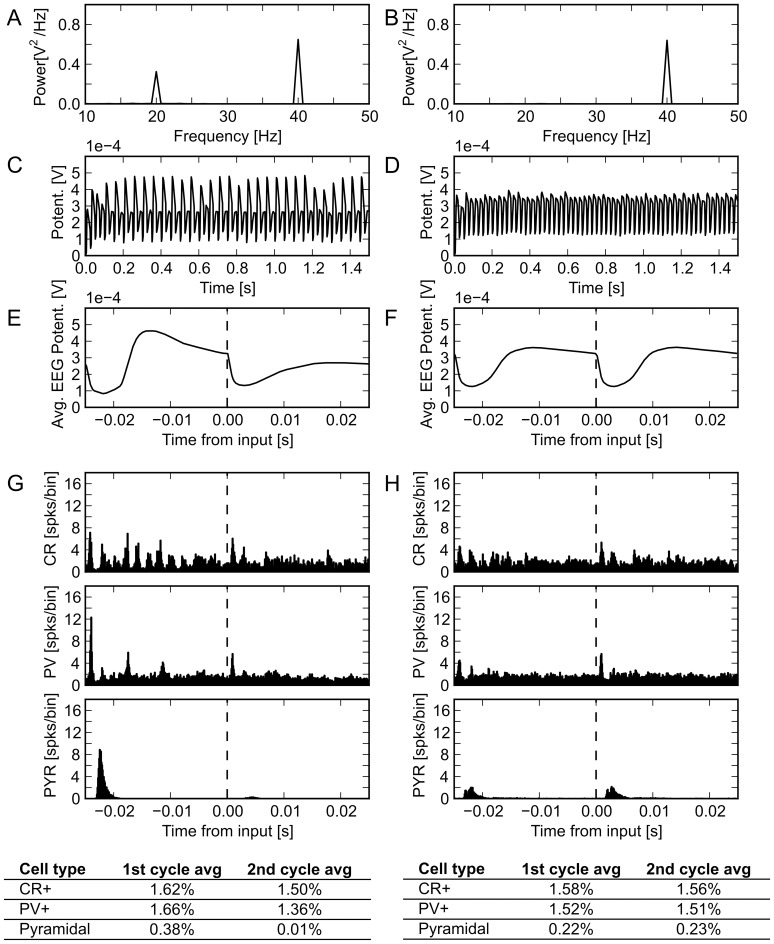
Response of schizophrenic model to 40 Hz drive. Simulated EEG traces in response to 40 Hz drive for primary schizophrenic point (left panels), and secondary schizophrenic point (right panels), as defined in text. (A, B) Power spectra of primary and secondary schizophrenic points, respectively. (C, D) Model produced EEG traces. (E, F) EEG, averaged over two consecutive cycles. (G, H) Spiking histogram and firing rates for individual neuron subtypes. Averages over sets of two consecutive cycles are shown. X-axis label applies to all three histograms. Potent  =  potential; spks  =  spikes; CR  =  calretinin positive cells; PV  =  parvalbumin positive cells; PYR  =  pyramidal cells.

What neural interactions caused the primary point, with a specific deficit in response to 40 Hz drive, to arise, and how did this differ from the secondary point? To answer this, we examined simulated EEG traces and histograms of spiking activity from both cases. Of note, for 40 Hz drive, the EEG traces of the primary point shows a depression of every other peak, effectively creating a mix of 20 and 40 Hz activity, and a decrease in the 40 Hz response (Fig 5). The spiking probability histograms for the primary and secondary points show averages over two cycles at a time, in an attempt to reveal differential contributions from inhibitory interneurons in alternating cycles. Notably, while both points produce a schizophrenic pattern of oscillatory activity when analyzed at the power spectrum level, there are clear differences in underlying neurophysiologic dynamics, as shown in Figure 5C and D. Panel C clearly shows alternating pyramidal cell activity across cycles; it also reveals a somewhat less marked alternation of PV+ cell activity, as well as modest cycle-to-cycle CR+ activity imbalance. Panel D (secondary point) shows a general damping down of pyramidal cell activity that is roughly constant across cycles, and little cycle-to-cycle variation in PV+ or CR+ activity.

### Simulation of Medication Effects

#### Negative controls:

An important goal of this work is to develop a model that can identify novel pharmacologic agents that can potentially treat the symptoms of schizophrenia. Such a model should also be capable of rejecting current medications known to have no known antipsychotic efficacy. Therefore, when applied to the schizophrenic model they should not produce normalization of oscillatory powers. These then serve as “negative controls”. We chose the test agents described below based on the following considerations: (a) Their neurophysiologic effects are well-characterized, and they can therefore be included in the model in a rigorous manner. (b) There is a published literature documenting their non-effectiveness in the illness. (c) There is a history of clinical use, and their effects on (control) subject EEG activity are known.

**Figure 6:**
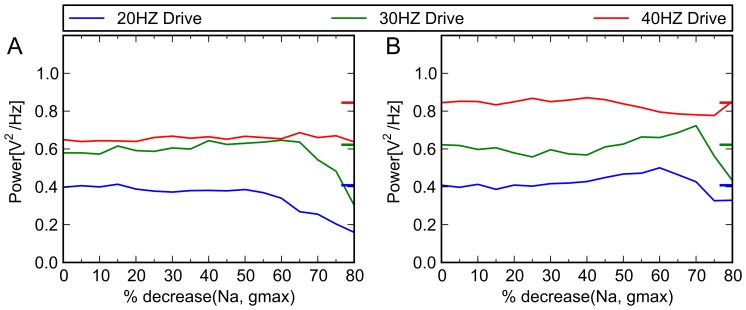
Simulated effects of phenytoin on control and schizophrenic models. The therapeutic dose range for phenytoin is 10–20 mg/L, (40–80 µmol/L [62]). Lampl et al [63] and others [64], [65] have shown that phenytoin concentrations in this range produce a decrease in Na^+^ channel conductance of between 34% and 50%. Above, x axis indicates percent reduction in conductance of Na^+^ channel, and y axis indicates the power in given frequency band of the model when driven at 20, 30 and 40 Hz. Colored tick marks on right border of graphs indicate oscillatory behavior characteristic of control subjects (A) Schizophrenic model. When we implement virtual medication doses, by gradually decreasing g_max_ of the Na^+^ channel, no ameliorative effect (i.e., specific increase in 40 Hz activity) was seen. (B) Control model. There are no known clinical studies that are precisely comparable to the experimental paradigm we have used—that is, studies of control subjects receiving phenytoin at various doses, who receive auditory click train stimulation at 20, 30, and 40 Hz. However, studies that have looked at resting EEG activity at therapeutically relevant doses have shown that it tends to increase 20 Hz activity [64], and have inconsistent effects on frequencies in the 30 Hz range [66], [67]; it was not seen to have a significant gamma band effect. Also, laboratory experiments using kainite-induced gamma oscillations in hippocampal slice preparations showed that therapeutic levels of phenytoin (50 μM) had no effect on gamma oscillations (p  =  0.05) [68]. When applied to our control model at the above doses, we achieve similar effects.

**Figure 7:**
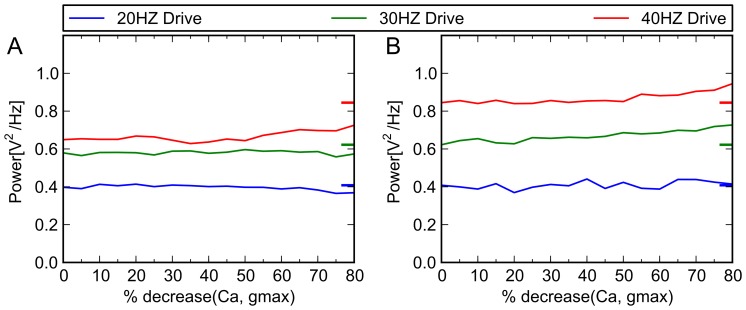
Simulated effects of nifedipine on control and schizophrenic models. This agent acts by blocking calcium channels. Electrophysiological studies have indicated that nifedipine can, depending on concentration, effectively decrease the slow inward Ca^++^ current by 50% or more. For illustrative purposes, we decreased calcium channel conductance by a maximum of 80%, in increments of 5%. Above, x axis indicates percent reduction in conductance of Ca^+^ channel, and y axis indicates oscillatory behavior (power in given frequency band) of model when driven at 20, 30 and 40 Hz. Colored tick marks on right border of graphs indicate oscillatory behavior characteristic of control subjects. **(A)** Schizophrenic model. When applied to the schizophrenic model, it did not show corrective effects, as expected. **(B)** Control model. For the 20 Hz range, clinical studies have shown no change under treatment with Ca^++^ channel blocker nimodipine [69], [70], or modest decreases in the relative power of this band [71]. For other frequency bands, general increases in resting EEG power [71] with treatment have been seen. Thus, simulation results are consistent with the clinical EEG literature.

**Figure 8:**
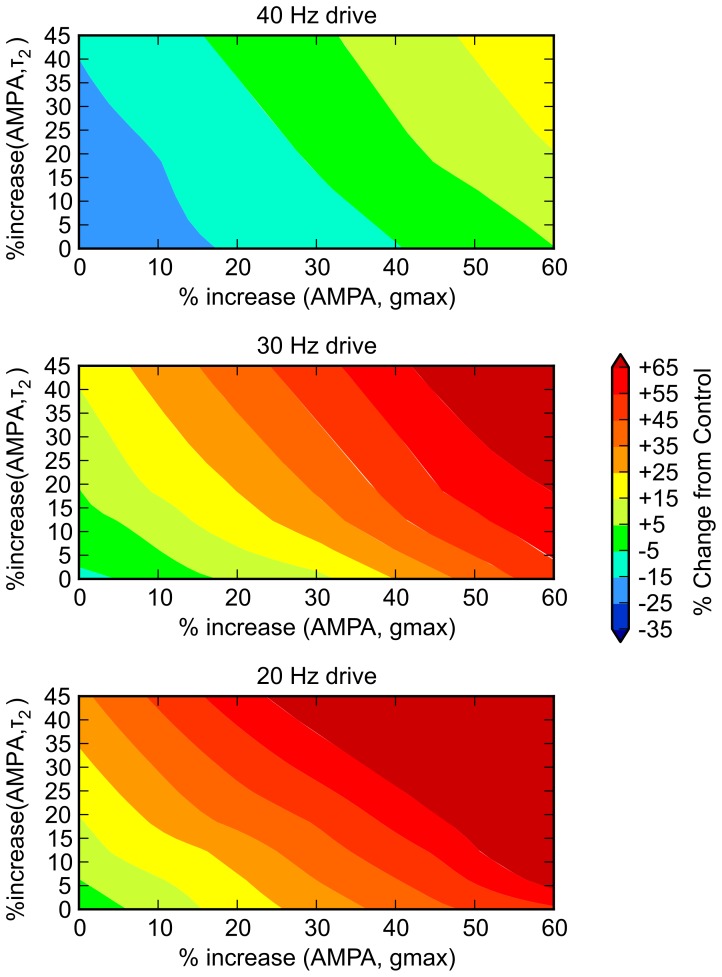
Ampakine application to schizophrenic model. Ampakines act by increasing maximum conductance of the AMPA channel (denoted by g_max_), increasing the delay time constant (denoted by τ_2_), or both. Moreover, various ampakines can differentially affect maximum amplitude and decay properties of the AMPA current [72]–[74]. To operationalize ampakine effects in model, we increased AMPA g_max_ by 0 to 60% (six gradations of 10%) and increased τ_2_ by 0 to 100% (five gradations of 20%), for a total of 30 iterations; we drove the model at 20, 30, and 40 Hz in each case. Color scale applies to all panels, and is identical to that of Figure 4, to facilitate comparison. Here, % change refers to change from the unaffected case; therefore, 0 represents re-equilibration. From the figure, it is clear that there is no particular effect on 40 Hz activity—within a reasonable range of parameter assumptions, a virtual ampakine that effectively normalized 40 Hz resonance would create supraphysiologic levels of 30 and 20 Hz activity. This is consistent with clinical findings: On theoretical grounds, it was felt that this class of drugs may have an ameliorative effects on schizophrenia and, a number of ampakines have been developed for clinical use (CX516 [Ampalex], CX717, CX691/Org24448 [Faramptor], and LY451395) [75]. However, clinical trials [76] have not borne out their effectiveness in patient populations.

For these trials, the primary point schizophrenic model, as defined above, is used as our test system. In separate trials, we apply the effects of phenytoin, an antiepileptic drug that has a specific effect at the Na+ channel (Fig 6); nifedipine, an antihypertensive that acts by blocking calcium channels (Fig 7); and ampakines, medications that allosterically bind to AMPA receptors and increase their activity [42]–[44], both by increasing maximum conductance and by increasing the decay time constant (Fig 8). In no case does the agent correct the 40 Hz deficit. Moreover, when applied to our unaffected model, they produce EEG changes comparable to those seen in the clinical literature. This serves as additional confirmation of the validity of the computational model.

#### Virtual medication trials:

Many experimental medications for schizophrenia act through one particular mechanism of action. However, it is possible that adjustment of a number of cellular level “levers” would be necessary to return the system to a healthy equilibrium state. We examined five such effects, applying each to the model individually, and in combinations with others. Broadly, these mechanisms fall into two categories: those that can be effected with currently known medications (discussed under AMPA g_max_, alpha2, and NMDA sections below); and those that, to the knowledge of these authors, cannot be implemented with any currently known agent (discussed under AMPA τ_2_ and CR+ projection below)—if effective, these would then represent potential targets for drug development efforts. The manner in which these were modeled is briefly described below, and are summarized in Table 1.

**Table 1:** Parameter ranges used for simulated medications.

Parameter	Description	Units	Range of Values	Incr
AMPA g_max_	conductance of AMPA channel	% increase	0, 20, 40, 60, 80	5
alpha_2_	conductance of GABA channel, alpha_2_ subtype	% increase	0, 15, 30, 45, 60	5
NMDA	conductance of NMDA channel^1^	% increase	0, 20, 40, 60, 80	5
AMPA τ_2_	decay time constant of AMPA channel	msec	1, 3, 5	3
CR proj	weight of projection of CR cells on postsynaptic targets	% increase	0, 20, 40, 60	4
total number of simulated trials: 1,500				

1 Resultant increase in intracellular Ca^++^ also induces LTP; the quantitative manner in which this is implemented is described in the text, and is in addition to the effect shown here.

Using the schizophrenic model, simulated medication trials were run, systematically varying model parameters through the ranges shown. A total of 3×5× 5×5×4  =  1,500 simulated trials were conducted. Incr  =  number of increments.

*AMPA g_max_.* The effect of drugs that boost AMPA current were modeled by increasing the maximum conductance (g_max_) of the AMPA synaptic current. We did this in increments of 20%, increasing g_max_ from 0% to 80%.

*Alpha_2_*. The experimental drug MK-0777 (also known as TPA-023) has partial agonist activity at GABA_A_ receptors, specifically acting at the α_2_ and α_3_ subtypes [45], and has shown partial effectiveness in treating some of the cognitive symptoms of schizophrenia [46]. These receptor subtypes are located on the initial segment of pyramidal cells, and are thought to be associated with the inhibitory projections of chandelier cells. While dissociation constants have been quantified [45], to our knowledge, MK-0777’s quantitative effect on GABA channel conductance has not been. Electrophysiological studies with mutant mice (knock-in mice selectively expressing GABA_A_ α_2_ , α_3_ , etc subtypes), has indicated that benzodiazepines can increase α_2_ and α_3_ conductance by as much as 50% [47]. Thus, to capture a plausible range of drug-induced conductance changes, we selectively increased the g_max_ of the GABA channels that synapse on the initial segment of pyramidal cells in increments of 15%, increasing g_max_ from 0 to 60%, in five gradations.

MK-0777 is one of the few cases in which a drug was tested in an experimental paradigm that involved schizophrenic patients and measurement of gamma band oscillations [46]. In this work, schizophrenic patients taking this drug showed a trend toward greater gamma band activity, which did not reach statistical significance at the p  =  0.05 level (their Figure 1, p. 1589-90). To ensure that our model system behaved similarly, we implemented an MK-0777 effect alone, and observed a very modest increase in 40 Hz resonance within certain dose ranges (data not shown), consistent with the experimental findings.

*NMDA*. NMDA boosting drugs, such as D-serine, have potential benefit both because they increase NMDA current, and because resulting intracellular calcium increases enhance long term potentiation (LTP). To model the former, we increased g_max_ of the NMDA conductance in increments of 20%, to a maximum 80% increase, in five gradations. Single cell modeling that we carried out suggested that the ratio of percentage NMDA conductance increase: overall intracellular calcium concentration increase was approximately 2:1. While it is known that increases in intracellular Ca^++^ concentration trigger LTP, their precise quantitative relationship remains uncertain [48]–[50]. Detailed modeling work by Shouval et al [51] suggests a ratio of approximately 62%: 29% (increase in calcium: degree of synaptic strengthening) (their Figure 1, p. 10832). Based on this, for every NMDA channel, for each 20% increase in NMDA channel conductance, we also increased the synaptic weight factor by 4.7%.

*AMPA τ_2_*. Modeling work described above suggests that significantly increasing the AMPA conductance decay time (τ_2_), in the manner of certain ampakines, does not improve model performance. However, in exploratory modeling work, we found that *decreasing* this parameter seemed to have positive effects (data not shown). Therefore, we used τ_2_ values of 1, 3 (control), and 5 msec.

*CR+ projections*. Exploratory runs of the schizophrenic model indicated that the calretinin cell projections (which impinge only on other interneurons) have a general quantitative modulatory role: increasing the weight of these projections tended to produce greater network activity overall (including 40 Hz oscillatory behavior) and decreasing them lead to generalized decreases in activity. Based on this, we adjusted upward the synaptic weight factor of the CR+ cells onto their postsynaptic targets, increasing it from 0 to 60%, in four gradations.

We ran each of the above effects alone, and in combination with all other effects, for a total of 5×5×5×3×4  =  1,500 trials (Table 1). For each trial, the model was driven at 20, 30, and 40 Hz, as described in our previous trials investigating schizophrenic pathology. To the extent that a simulated medication specifically increased 40 Hz power response to 40 Hz drive, it was considered effective. That is, if a treated schizophrenic model exactly replicated control model behavior, it would receive a score of 1.0; the score was decreased to the extent that it departed from this. Trials that did not produce 20 Hz and 30 Hz power within 10% of control were marked as failed trials, and received a score of 0. Thus, a simulated drug that boosted all frequencies indiscriminately would not be considered effective.

**Figure 9:**
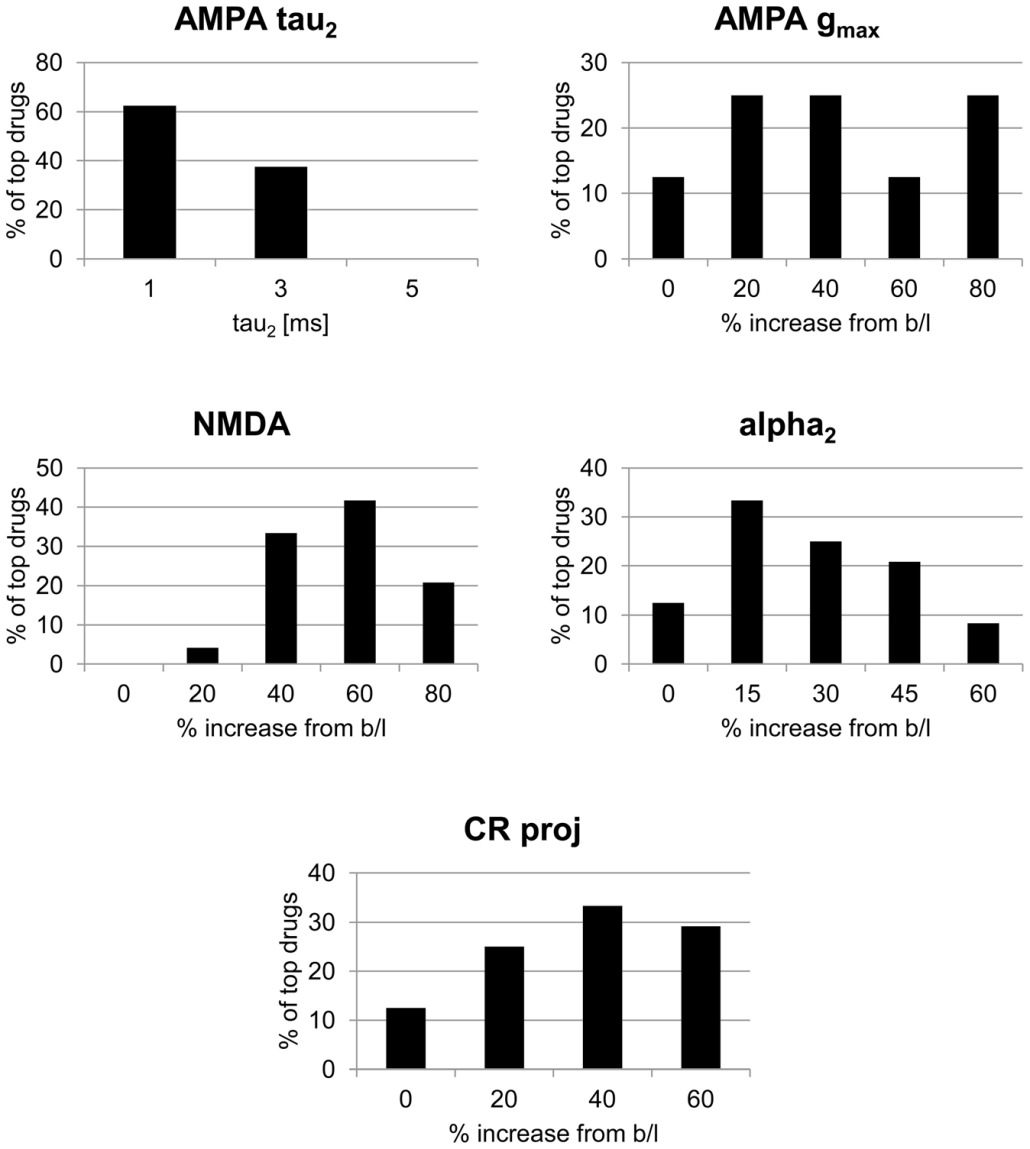
Model response to simulated medication effects. Breakdown, by mechanism of action, of top 24 most effective simulated drugs (those scoring 0.9 or higher). Y axis indicates fraction of top 24 drugs having the quantitative alteration shown for the given mechanism (e.g., slightly greater than 60% of the top drugs had an AMPA τ_2_ value of 1 ms). Titles correspond to mechanisms of action described in text. Baseline value for AMPA τ_2_ is 3 ms. b/l  =  baseline.

In total, 97 virtual medications, or 6.5% of the 1,500 tried, produced non-zero scores. 24 received scores 0.90 or higher. The characteristics of these drugs are shown via histograms in Figure 9.

It can be seen that of these top performing medications, many decreased AMPA τ_2_ and modestly increased NMDA activity. Clearly, a number of these effects may have interacted to produce desirable outcomes. To understand this at a fine grained level, we ran a three-way analysis of variance on the 97 scored virtual medications; results are shown in Table 2, with effects showing significance at a level of p < 0.001 indicated. Of note, (decreasing) AMPA τ_2_ emerged as a significant effect, alone and in combination. A very significant interaction between AMPA τ_2_ and CR+ projection strength also emerged.

**Table 2:** Analysis of variance of model response to drug effects.

Simulated drug effect	F value	p value	
alpha_2_	0.01	0.916	
AMPA g_max_	1.76	0.185	
AMPA τ_2_	25.14	6.00E-07	*
NMDA	21.17	4.55E-06	*
CR proj	0.35	0.554	
alpha_2_:AMPA g_max_	0.92	0.338	
alpha_2_:AMPA τ_2_	1.23	0.268	
AMPA g_max_:AMPA τ_2_	13.43	2.56E-04	*
alpha_2_:NMDA	6.52	0.011	
AMPA g_max_:NMDA	5.24	0.022	
AMPA τ_2_:NMDA	0.01	0.914	
alpha_2_:CR proj	1.78	0.182	
AMPA g_max_:CR proj	10.44	0.001	
AMPA τ_2_:CR proj	68.18	3.29E-16	*
NMDA:CR proj	1.91	0.167	
alpha_2_:AMPA g_max_:AMPA τ_2_	0.84	0.358	
alpha_2_:AMPA g_max_:NMDA	0.48	0.490	
alpha_2_:AMPA τ_2_:NMDA	1.53	0.217	
AMPA g_max_:AMPA τ_2_:NMDA	1.57	0.211	
alpha_2_:AMPA g_max_:CR proj	0.37	0.545	
alpha_2_:AMPA τ_2_:CR proj	4.19	0.041	
AMPA g_max_:AMPA τ_2_:CR proj	1.11	0.292	
alpha_2_:NMDA:CR proj	2.69	0.101	
AMPA g_max_:NMDA:CR proj	4.21	0.040	
AMPA τ_2_:NMDA:CR proj	13.34	2.70E-04	*

* p < 0.001

Using the wellness metric (see text), as the outcome variable, 97 of 1,500 simulated medications produced non-zero values. ANOVA of this output, using parameters for drug effects as factors, is shown. Highly significant effects (p < 0.001) are indicated. CR proj  =  CR+ projection.

## Discussion

It is clear that antipsychotics with fundamentally new mechanisms of action are needed. A primary factor hindering their development has been a lack of well specified, detailed models indicating how the myriad cellular level abnormalities that have been identified experimentally interact to create symptoms. Our work represents an initial attempt to address these questions. Models like ours may allow a process of drug development in which the “target” is not necessarily a single neural entity that is abnormal, but rather a system level behavior that has become dysfunctional. It may be possible to treat the illness by acting on biological entities that are not directly causative, but that can serve to re-equilibrate the system.

### Mechanistic Implications

While we acknowledge that 40 Hz oscillatory deficit is not a “classical” symptom of schizophrenia, we felt it was a highly appropriate outcome measure for this computational study for two reasons. First, given the likely importance of gamma band activity in subserving perceptual binding within and across sensory modalities and in cognition generally [18], and the core schizophrenic symptoms of compromised reality testing and hallucinations, a strong argument can be made that the gamma band biomarker is tapping into an important characteristic of the illness. Second, a growing body of clinical work suggests that it may represent an important endophenotypic marker of the disease. In a recent review of this literature [52], it was shown that across all frequencies (theta [4–7 Hz], alpha [8–12 Hz], beta [12–30 Hz], and gamma) and testing paradigms (steady state evoked potentials, induced responses, evoked responses, and resting state measurement), studies that showed the most robust and consistent evidence for a schizophrenic patient-specific phenomenon were those looking at steady state evoked responses in the gamma band. Because endophenotypes—as opposed to complex clinical phenotypes—may be more closely related to the genetic underpinnings of the disease, a focus on these markers may be extremely valuable in elucidating etiology and informing treatments [53].

Our modeling suggests that in the hippocampal etiology of schizophrenia, neural level abnormalities are perhaps not simply additive—that is, that more pathology, regardless of type, necessarily creates more illness. Rather, it appears that there may be one or more discrete *combinations* of abnormalities that give rise to the decreased gamma band activity that is associated with the illness. Of note, both sets of pathology we identified were characterized by co-occurring modest reduction in spine density, as well as reductions in NMDA functionality. Neither of these lesions alone, even occurring at extreme levels, was seen to be associated with schizophrenia-like model behavior. It appeared that particular combinations of GABA system lesions could lead to a specific, and modest, lessening of 40 Hz response; but no combinations of GABA lesions alone resulted in a pattern that was quantitatively similar to the schizophrenic dysfunction seen in the literature.

Significantly, the two clusters we identified were associated with dissimilar underlying neural dynamics. The most pronounced one (the primary point) showed a highly regular “beat skipping” quality, which created increased 20 Hz resonance in addition to decreased 40 Hz activity. A similar mechanism was seen in a previous modeling study [54]. This is particularly significant because that study implemented a very different mechanism—a lengthening of the decay time constant of the projections of PV+ interneurons—to generate similar behavior. This raises the possibility that this behavior may be an important mechanistic trait associated with the illness, at least in particular brain areas, and that different sets of cellular level abnormalities can give rise to it.

The other schizophrenic combination (the secondary point) we identified did not show this behavior, but appeared to arise from a dampening of 40 Hz behavior generally. This is consistent with the apparent inconsistencies in clinical research, in which some studies show a higher 20 Hz response [55], and many studies did not [17], [41] among schizophrenic patients. Schizophrenia’s extreme heterogeneity has always been puzzling. This modeling work raises the possibility that different subtypes are associated with particular sets of neural abnormalities.

Post-mortem and other wet lab research methodologies tend to be very labor intensive, and investigating all combinations of possible neural abnormalities in large numbers of samples is not practical. The type of modeling study described here could be used as a guide, or hypothesis-generating tool, for laboratory research. Moreover, if clinical information is known about the tissue source (which is usually the case), the hypothesis that particular clusters of neural abnormalities correspond to particular subtypes can be tested.

### Treatment Implications

Many traditional drug discovery efforts have involved identifying a cellular level abnormality associated with an illness, and creating an agent to counteract that particular deficiency. These efforts have not been entirely successful in the case of schizophrenia, and the modeling work here presents an alternative approach. As a test system, we used the primary point schizophrenia model, as described above. First, medications with no know antipsychotic efficacy were introduced to the schizophrenic model, to ensure that the model identified them as such. This is admittedly a “low bar”, but any test system to identify potentially effective agents should, at a minimum, be able to reject those that are clinically known to be inactive. Then, we carried out a series of 1,500 virtual medication trials, using five different potential drug mechanisms.

Perhaps the most surprising outcome of these simulated drug trials was the model’s prediction that medications that decrease the decay time constant of the AMPA channel would be potentially effective agents. This was apparent in looking at “wellness metric” data descriptively; it also emerged on two and three way analyses of variance. Of the five virtual drugs with highly significant p values, four involved an AMPA τ_2_ effect. This and other computational studies [54] have suggested that lingering or “blurring” of inhibitory processes may prevent the system from attuning to the relatively fast 40 Hz input drive. To the extent that a reduction of AMPA τ_2_ causes a sharpening of signaling, this could be beneficial.

Also, there were marked interactions *between* effects. Notably, the ANOVA showed a very weak single factor CR+ projection strength effect, but an extremely strong interactive effect with decreases in AMPA τ_2_ (the most robust interaction of all combinations tested). There was a very strong interaction between AMPA τ_2_ decrease and AMPA g_max_ as well. Increasing AMPA g_max_ and CR+ projection are similar in that alone, they would have the effect of increasing excitatory activity generally. It is therefore not surprising that ANOVA revealed these particular interactions—these combinations may result in an increased magnitude of a more “precise” signal.

The implications of these findings are threefold: First, it is possible that a given unsuccessfully tested mechanism (e.g. increasing AMPA conductance via ampakines) is not incorrect, strictly speaking, but rather incomplete—that is, in combination with other cellular level effects, amelioration of symptoms could be achieved. However, because of system complexity, it is difficult to determine *a priori*, based on deductive reasoning alone, which particular combination of “levers” could lead to a re-equilibration. Modeling can help identify the particular combination of mechanisms that will constitute effective medications.

Second, based on the existing literature, CR+ interneurons have not been implicated as a cause of schizophrenia [56]-[58], nor were they altered in our model to render it schizophrenic. The same is true of the decay time constant of the AMPA conductance. Nonetheless, altering these features helped to re-equilibrate the system, and return it to its control state. This implies that the search for effective antipsychotics need not be limited to neural elements that have been demonstrated in postmortem or other wet lab work to be abnormal in the illness.

Finally, this model makes specific, testable hypotheses. To our knowledge, there is no existing drug that specifically increases the weight of CR+ post-synaptic projections or increases the conductance at these synapses, or that specifically decreases the APMA decay time constant. This work suggests that medications that incorporate these effects may be efficacious for schizophrenia.

## Supporting Information

Table S1 **Summary of compartmental parameters for neuronal models.**
(DOCX)

Table S2 **Model parameters by cell type and subcellular location.**
(DOCX)

Table S3 **Synaptic channel parameters.**
(DOCX)

Table S4 **Model connectivity. Model is connected randomly using the indicated probabilities for each connection type.**
(DOCX)

Table S5 **Illness metric parameters.**
(DOCX)

Text S1 **Supplemental text.**
(DOCX)
