# Supplementary material for: Development of Antipsychotic Medications with Novel Mechanisms of Action Based on Computational Modeling of Hippocampal Neuropathology
Source: PLoS One. 2013 Mar 19;8(3):e58607. doi: 10.1371/journal.pone.0058607 (PMC3602393; doi:10.1371/journal.pone.0058607)
Supplement: Table S1 — Summary of compartmental parameters for neuronal models. (DOCX) [file pone.0058607.s001.docx]

**Table S1.** Summary of compartmental parameters for neuronal models.

| **Parameter** | **Definition** | **Value** |
| --- | --- | --- |
| $E_{m}$ | Resting membrane potential | -60.0 mV |
| $C_{M}$ | Specific membrane capacitance | Vary by compartment and cell type  (see below). |
| $R_{M}$ | Specific membrane resistance |  |
| $R_{A}$ | Specific axial resistance |  |
| $R_{a}$ | Axial resistance | $R_{a}=4lR_{A}/\left(\pi d^{2}\right)$ |
| $C_{m}$ | Membrane capacitance | $C_{a}=\pi ldC_{M}$ |
| $R_{m}$ | Membrane resistance | $R_{a}=R_{M}/\left(\pi ld^{2}\right)$ |
