# Supplementary material for: Development of Antipsychotic Medications with Novel Mechanisms of Action Based on Computational Modeling of Hippocampal Neuropathology
Source: PLoS One. 2013 Mar 19;8(3):e58607. doi: 10.1371/journal.pone.0058607 (PMC3602393; doi:10.1371/journal.pone.0058607)
Supplement: Table S2 — Model parameters by cell type and subcellular location. (DOCX) [file pone.0058607.s002.docx]

**Table S2.** Model parameters by cell type and subcellular location.

| **Parameter** | **Pyramidal Cells** | | | **Interneurons** | | |
| --- | --- | --- | --- | --- | --- | --- |
|  | Soma | Axonal Initial Segment | Dendrite | Soma | Axonal Initial Segment | Dendrite |
| *C_M_* [µF/cm^2^] | 0.75 | 0.75 | 1.5 | 0.75 | 0.75 | 0.75 |
| *R_M_* [KΩcm^2^] | 50 | 1.0 | 25 | 50 | 1.0 | 50 |
| *R_A_*[KΩcm] | 0.2 | 0.1 | 0.2 | 0.2 | 0.1 | 0.2 |
