# Supplementary material for: Development of Antipsychotic Medications with Novel Mechanisms of Action Based on Computational Modeling of Hippocampal Neuropathology
Source: PLoS One. 2013 Mar 19;8(3):e58607. doi: 10.1371/journal.pone.0058607 (PMC3602393; doi:10.1371/journal.pone.0058607)
Supplement: Table S3 — Synaptic channel parameters. (DOCX) [file pone.0058607.s003.docx]

**Table S3.** Synaptic channel parameters.

| **Parameter** | **Definition** | **Value** |
| --- | --- | --- |
| $g_{max, NMDA}$ | Maximum conductance of NMDA | 160x10^-12^ S |
| $g_{max, GABA_{A}}$ | Maximum conductance of $GABA_{A}$ | 40x10^-12^ S |
| $g_{max, AMPA}$ | Maximum conductance of AMPA | 80x10^-12^ S |
| $\tau_{\text{1,}NMDA}$ | First time constant of NMDA | 0.080 S |
| $\tau_{\text{2,}NMDA}$ | Second time constant of NMDA | 0.000670 S |
| $\tau_{\text{1,}GABA_{A}}$ | First time constant of GABA | 0.003 S |
| $\tau_{\text{2,}GABA_{A}}$ | Second time constant of GABA | 0.008 S |
| $\tau_{\text{1,}AMPA}$ | Fist time constant of AMPA | 0.003 S |
| $\tau_{\text{2,}AMPA}$ | Second time constant of AMPA | 0.003 S |
