# Supplementary material for: Development of Antipsychotic Medications with Novel Mechanisms of Action Based on Computational Modeling of Hippocampal Neuropathology
Source: PLoS One. 2013 Mar 19;8(3):e58607. doi: 10.1371/journal.pone.0058607 (PMC3602393; doi:10.1371/journal.pone.0058607)
Supplement: Table S4 — Model connectivity. Model is connected randomly using the indicated probabilities for each connection type. (DOCX) [file pone.0058607.s004.docx]

**Table S4.** Model connectivity. Model is connected randomly using the indicated probabilities for each connection type.

| Cell type | Number of Cells | Post Synaptic target | Post-Synaptic  receptors | Connectivity  probability | Connection Weights |
| --- | --- | --- | --- | --- | --- |
| Pyramidal | 160 | Pyramidal dendrites  Basket dendrites  Chandler dendrites  CR+ dendrites | NMDA and  AMPA | 5%  21%  21%  27% | 10  10  10  10 |
| Basket | 30 | Pyramidal proximal dendrites and soma | GABA | (13%)* | 60 |
| Chandler | 30 | Pyramidal IS | GABA | (13%)* | 120 |
| CR | 20 | Basket dendrites  Chandler dendrites  CR+ dendrites | GABA | 100%  100%  100% | 30  10  30 |

* Pyramidal cells receive projections from 13% of basket cells and 13% of chandelier cells.
