# Supplementary material for: Development of Antipsychotic Medications with Novel Mechanisms of Action Based on Computational Modeling of Hippocampal Neuropathology
Source: PLoS One. 2013 Mar 19;8(3):e58607. doi: 10.1371/journal.pone.0058607 (PMC3602393; doi:10.1371/journal.pone.0058607)
Supplement: Table S5 — Illness metric parameters. (DOCX) [file pone.0058607.s005.docx]

**Table S5.** Illness metric parameters.

| Drive Frequency[Hz] | $\delta_{1}$ | $\delta_{2}$ | $P\left(S_{f}\right)$ |
| --- | --- | --- | --- |
| 20 | 0.075 | 0.075 | 0.00 |
| 30 | 0.075 | 0.075 | 0.00 |
| 40 | 0.26 | 0.26 | -0.26 |
