# Supplementary material for: Development of Antipsychotic Medications with Novel Mechanisms of Action Based on Computational Modeling of Hippocampal Neuropathology
Source: PLoS One. 2013 Mar 19;8(3):e58607. doi: 10.1371/journal.pone.0058607 (PMC3602393; doi:10.1371/journal.pone.0058607)
Supplement: Text S1 — Supplemental text. (DOCX) [file pone.0058607.s006.docx]

**SUPPLEMENTAL TEXT**

*Development of antipsychotic medications with novel mechanisms of action based on computational modeling of hippocampal neuropathology*, by Peter J. Siekmeier and David P. vanMaanen

***Individual Neuron Models***

The hippocampal model consists of a total of 240 simulated neurons: 160 pyramidal cells, and interneurons of three subtypes (30 basket cells, 30 chandelier cells, and 20 calretinin-positive [CR+], or interneuron projecting, cells). For pyramidal cells and interneurons, we used the 64 compartment model described by Traub et al [[1](#_ENREF_1)], and the 46 compartment model of Traub and Miles [[2](#_ENREF_2)], respectively.

Each compartment of the individual neuron models is represented as follows:

| $C_{m}\frac{dV_{m}}{dt}=\frac{\left(E_{m}-V_{m}\right)}{R_{m}}+\frac{\left(V'_{m}-V_{m}\right)}{R_{a}}+I_{syn}+I_{ionic}$, | (1) |
| --- | --- |

where *V_m_* is the transmembrane potential and *V'_m_* is the transmembrane potential of the adjacent compartment, *C_m_* is the membrane capacitance, *E­_m_* is the resting membrane potential, *R_m_* is the membrane resistance, *R­_a_* is the axial resistance, and *I_syn_* and *I_ionic_* terms are the sums of current from voltage-dependent ionic channels and synaptic channels, respectively; values for the constants used in the neuron models are shown in Supplemental Tables S1 and S2.

The individual ionic channel currents take the form

| $I_{channel}=G_{channel}\left(E_{channel}-V_{m}\right)$, | (2) |
| --- | --- |

where *I_channel_* is the current of an ionic channel, *E_channel_* is the reversal potential of that channel, and *G_channel_* is the variable conductance of that channel. Conductances in this model are those used in the aforementioned articles [[1](#_ENREF_1),[2](#_ENREF_2)].

Synaptic currents were also included. AMPA and GABA_A_ currents take the form of Equation 3, where the reversal potential is 0 mV for NMDA, 45 mV for AMPA and -82 mV for GABA. Conductances were assumed to obey a dual exponential function, as follows:

| , | (3) |
| --- | --- |

where$H\left(t-t_{n}\right)$is the Heaviside function, *A* is a normalization factor such that the maximum conductance is g_max_,$\tau_{1}$and$\tau_{2}$are time constants, and *W* is the weight factor for the particular connection. The NMDA channel takes the form shown in Equation 3, with modifications to instantiate magnesium block behavior [[3](#_ENREF_3)]. All parameters for synaptic connections are given in Supplemental Table S3.

***Connectivity and Stimulation***

Cell to cell connectivity was based on hippocampal modeling previously described by the author [[4](#_ENREF_4)]. In summary, the following assumptions were made: (1) Pyramidal cells (PCs) project very sparsely to

other pyramidal cells. (2) Basket cells densely innervate somata and proximal dendrites of PCs. (3) Chandelier cells synapse only on axonal initial segments of PCs. (4) Calretinin cells project densely to other interneurons; they do not innervate PCs. All connectivity parameters are presented in Supplemental Table S4.

For pyramidal projections, postsynaptic receptors were divided between AMPA and NMDA synapses. Based on an extensive review of the hippocampal neuroanatomy literature, PC to PC projections were taken to be 10% NMDA and 90% AMPA [[5](#_ENREF_5),[6](#_ENREF_6)], and PC to interneuron projections were 40% NMDA and 60% AMPA [[7-9](#_ENREF_7)]. All interneuron projections formed GABA synapses.

The model was driven by spike train at the given test frequency (20, 30, or 40 Hz). The drive was delivered to all pyramidal cells with a dedicated AMPA-type receptor, and 50% of interneurons. This was based on anatomical data which indicates that incoming projections impinge on relatively fewer interneurons, compared with PCs [[10](#_ENREF_10)].

In the context of the entire hippocampal formation, our model represents a very small piece of tissue; thus, the amount of innervation received by a given cell is considerably smaller than that received by a hippocampal cell *in vivo.* For example, actual CA1 PCs receive about 30,000 excitatory and 1,700 inhibitory inputs [[11](#_ENREF_11)]; the inputs received by the cells in our model were orders of magnitude lower than this. To compensate, we multiplied the synapses of the model by a weight factor of 10. Also, a given basket cell projects to a PC and forms a dense nexus of connections around the receiving cell’s soma and proximal dendrites. Similarly, chandelier cells project densely to IS, and because of this, have a particular pronounced effect on PC output. Weight multipliers for these two cell types are increased to reflect these facts, as indicated in Supplemental Table S4. For model input drive, the multiplier was 500.

***Simulated EEG***

To compare the behavior of our system to the experimental data, it was necessary to calculate a simulated EEG for the system. Biologically, an EEG signal is produced by current flowing through ion and synaptic channels, thus creating a changing electric potential measured at the scalp. According to the Nunẽz equation [[12](#_ENREF_12)], this potential is found by

| , | (4) |
| --- | --- |

where$\Phi$is the potential measured at a point $\vec{r}$at time, *t,* and *R_i_* is the distance, through a medium with conductivity,$\sigma$ between the current,$I_{n}$ and the point (the electrode).

For the simulation, we assumed that the tissue modeled was of negligible spatial distribution; distance R is assumed to be 6.8 mm, the average skull thickness [[13](#_ENREF_13)]. We used an estimated median of value for conductivity of the brain of 0.251 S/m [[14](#_ENREF_14)]. As in previous studies, we consider only the contribution of the excitatory channels, as this is felt to be the main contributor to EEG activity. Therefore, the EEG is calculated in our study by

| $\Phi\left(t\right)=\frac{1}{4\pi\sigma R}\sum_{n=1}^{N} I_{n}$, | (5) |
| --- | --- |

Where *Φ* is the potential measured at a single point outside of the skull, and *N* is the total number of excitatory synaptic channels.

***Computing Details***

The models described above were implemented using the General Neural Simulation System (GENESIS) version 2.3 [[15](#_ENREF_15)], using programs written in C++ with MPI to conduct parameter searches. The backward Euler integration method, as implemented in standard GENESIS, was used to solve the above equations with a 0.1 ms time step. All modeling was carried out on a 72-processor dedicated Beowulf computer cluster (PSSC Labs/Professional Service Supercomputers, Lake Forest, CA), running under RHEL5 64-bit Linux operating system within the Laboratory for Computational Neuroscience at McLean Hospital.

***Illness Metric***

We created an “illness metric” as a quantitative definition of schizophrenia for this study, based on the driven frequency abnormalities observed in the disease. The metric varies from 0 to 1 with 1 being the most schizophrenic-like. The metric is defined as:

| $I\left(q\right)=\prod_{f=\text{20,30,40}} l_{f}\left(\rho_{f}\left(q\right)\right)$, | (6) |
| --- | --- |

where$I\left(q\right)$ is the illness metric at the point q in the parameter space,$l_{f}$is the individual illness metric at a drive frequency of, *f*, and $\rho_{f}$is the height of power spectrum peak at that frequency. The individual frequency metrics measure the contribution of each frequency to the final metric. For drive frequencies of 20 Hz and 30 Hz, this metric is simply a pass or fail test. Specifically:

| $l_{f}\left(\rho_{f}\right)=\left\{\begin{matrix} 1 if P\left(S_{f}\right)-\delta_{\text{2,}f}\leq P\left(\rho_{f}\right)\leq P\left(S_{f}\right)+\delta_{1,f} \\ 0 otherwise \end{matrix}\right\}$, | (7) |
| --- | --- |

where$P\left(S_{f}\right)$is the percent change from control of the ideal schizophrenic power and $\delta_{\text{1,}f}$and $\delta_{\text{2,}f}$are arbitrary constants used to describe the acceptable range for each frequency. This gating effect for 20 and 30 Hz simply eliminates all points which fall in a non-biologically realistic range. For the 40 Hz drive frequency, a graded function is used to rank the effects. This emphasizes the important role that gamma band oscillations are thought to play in schizophrenia. The function used for the 40 Hz drive frequency is:

| $l_{40}\left(\rho_{40}\right)=\left\{\begin{matrix} 0 if P\left(\rho_{40}\right) < P\left(S_{40}\right)-\delta_{\text{2,} 40} \\ 1-\frac{\left(1-a\right)}{\delta_{\text{2,} 40}}\left(P\left(\rho_{40}\right)-P\left(S_{40}\right)\right) if P\left(S_{40}\right)-\delta_{\text{2,} 40} \leq P\left(\rho_{40}\right) < P\left(S_{40}\right) \\ 1+\frac{\left(1-a\right)}{\delta_{\text{1,} 40}}\left(P\left(\rho_{40}\right)-P\left(S_{40}\right)\right) if P\left(S_{40}\right) \leq P\left(\rho_{40}\right) \leq P\left(S_{40}\right)+\delta_{1, 40} \\ 0 if P\left(\rho_{40}\right) > P\left(S_{40}\right)+\delta_{2, 40} \end{matrix}\right\}$ | (8) |
| --- | --- |

where $a$ is the lowest score possible for a peak within bounds (0.01) . This metric is controlled by several constants, as shown in Supplemental Table S5.

***References***

1. Traub RD, Jefferys JG, Miles R, Whittington MA, Toth K (1994) A branching dendritic model of a rodent CA3 pyramidal neurone. Journal of Physiology (London) 481: 79-95.

2. Traub RD, Miles R (1995) Pyramidal cell-to-inhibitory cell spike transduction explicable by active dendritic conductances in inhibitory cell. Journal of Computational Neuroscience 2: 291-298.

3. Zador A, Koch C, Brown T (1990) Biophysical model of a Hebbian receptor. Proceedings of the National Academy of Sciences of the USA 10: 6718-6722.

4. Siekmeier PJ (2009) Evidence of multistability in a realistic computer simulation of hippocampus subfield CA1. Behavioural Brain Research 200: 220-231.

5. Nicholson D, Trana R, Katz Y, Kath W, Spruston N, et al. (2006) Distance-dependent differences in synapse number and AMPA receptor expression in hippocampal CA1 pyramidal neurons. Neuron 50: 431-442.

6. Nusser Z (2000) AMPA and NMDA receptors: similarities and differences in their synaptic distribution. Current Opinion in Neurobiology 10: 337-341.

7. Sah P, Hestrin S, Nicoll R (1990) Properties of excitatory postsynaptic currents recorded in vitro from rat hippocampal interneurones. Journal of Physiology 430: 605-616.

8. Baude A, Nusser Z, Molnar E, McIlhinney R, Somogyi P (1995) High-resolution immunogold localization of AMPA type glutamate receptor subunits at synaptic and non-synaptic sites in rat hippocampus. Neuroscience 69: 1031-1055.

9. Nyiri G, Stephenson F, Freund T, Somogyi P (2003) Large variability in synaptic N-methyl-D-aspartate receptor density on interneurons and a comparison with pyramidal-cell spines in the rat hippocampus. Neuroscience 119: 347-363.

10. Binzegger T, Douglas R, Martin K (2004) A quantitative map of the circuit of cat primary visual cortex. Journal of Neuroscience 24: 8441-8453.

11. Megias M, Emri ZS, Freund TF, Gulyas AI (2001) Total number and distribution of inhibitory and excitatory synapses on hippocampal CA1 pyramidal cells. Neuroscience 102: 527-540.

12. Nunez PL (1981) Electric fields of the brain : The neurophysics of EEG. New York: Oxford University Press. 484 p.

13. Li H, Ruan J, Xie J, Wang H, Liu W (2007) Investigation of the critical geometric characteristics of living human skulls utilizing medical image analysis techniques. International Journal of Vehicle Safety 2: 345-367.

14. Huang M, Song T, Hagler D, Podgorny I, Jousmaki V, et al. (2007) A novel integrated MEG and EEG analysis method for dipolar sources. Neuroimage 37: 731-748.

15. Bower JM, Beeman D (1998) The Book of GENESIS: Exploring Realistic Neural Models with the GEneral NEural SImulation System. Santa Clara, CA: Springer-VerlagTelos.
